# TACC3 in personalized medicine

**DOI:** 10.18632/oncoscience.229

**Published:** 2015-08-31

**Authors:** Stephen Murata, Loredana Campo, Eun-Kyoung Breuer

**Affiliations:** Department of Radiation Oncology, Stritch School of Medicine, Loyola University Chicago, Maywood, IL, USA

**Keywords:** TACC3, DNA damage response

Cells are constantly exposed to endogenous and exogenous DNA damage events. In order to confront DNA damage and to maintain genomic integrity, cells have evolved a fine-tuned network of cellular pathways, collectively known as the DNA damage response (DDR). The broad components of DDR include surveillance mechanisms, cell cycle checkpoints, DNA repair pathways, and apoptotic programs. Interestingly, the very deficiencies in DDR that lead to cell death, genomic instability, and tumorigenesis, are also the very mechanisms that can be harnessed for cancer's own demise. Such vulnerabilities manifest as deregulated DDR-associated pathways that, with further elucidation, can become prognostic and predictive biomarkers and therapeutic targets in personalized cancer therapy.

Transforming Acidic Coiled-Coil 3 (TACC3) is acknowledged for its canonical role in centrosome-microtubule spindle dynamics, and its deregulation has been linked to a variety of human cancers, including breast, colon, liver and lung cancers [1]. Also, high TACC3 has been shown to be strongly associated with poor survival in breast and lung cancer patients [1]. Interestingly, p53-deficient mouse with conditional knockout of *Tacc3* showed a remarkable thymus lymphoma regression [2]. Given these associations, it is imperative to clarify the precise mechanistic contribution of TACC3 to tumorigenesis and cancer development.

Previously, we demonstrated that TACC3 plays a pivotal role in the process of epithelial-mesenchymal transition (EMT), a key step of tumor progression and metastasis, by promoting phosphoinositide 3-kinase (PI3K)/Akt and extracellular signaling related kinase (ERK) signaling [3]. In our most recent study, we found that high TACC3 induces spontaneous DNA damage and impairs G2/M checkpoint function and repair of DSBs (both homologous recombination [HR] and non-homologous end joining [NHEJ]), thus increasing the frequency of chromosomal aberrations, at least in part, by TACC3-mediated negative regulation of Ataxia telangiectasia mutated (ATM) [1]. Given TACC3's emerging role in DDR regulation, we then sought to investigate TACC3's predictive value for hypersensitivity to radiation and poly(ADPribose)polymerase (PARP) inhibitor treatment. Unsurprisingly, high TACC3 levels confer cellular hypersensitivity to radiation and the PARP inhibitors Olaparib (AZD2281) and NU1025 [1].

In spite of the emerging link between high TACC3 and impaired DDR, unanswered questions need to be uncovered to have a complete knowledge of TACC3 functionality in human cancer. For example, the fact that re-expression of ATM did not completely rescue high TACC3-mediated DNA damage, suggests that there are other mechanisms unaccounted for that might contribute to TACC3-mediated genomic instability. Since Aurora Kinase A (AurA) acts as an upstream of TACC3 [4], and because both TACC3 and Aurora Kinase A disrupt normal cellular response to DNA damage, although TACC3 affects both Chk1 and Chk2 [1] while AurA only affects Chk1 [5], it would be interesting to clarify whether TACC3-mediated disruption of DDR occurs in an AurA-dependent manner. Additionally, a more robust understanding of the upstream regulation of TACC3 is required; this may shed light on the etiology of TACC3 upregulation in various cancers, and provide a starting point for thinking about cancer prevention. Meanwhile, since TACC3 is actually downregulated in a certain subset of cancers [6], it remains unclear if TACC3 is a tumor suppressor, an oncogene, or both. In any case, it is clear that TACC3 deregulation is associated with cancer.

While knowledge gaps of TACC3's role in cancer still remain, the clinical-translational significance of TACC3 is becoming increasingly clear. The distribution of TACC3 at the interface of the mitotic spindle-assembling machinery, vital for tumor survival and progression, makes TACC3 a superior therapeutic target for anti-cancer drugs precisely designed to inhibit the mitotic spindle-microtubule of cancer with aberrant TACC3, without interfering with the microtubules activity in non-dividing cell. Furthermore, we discovered that depletion of TACC3 renders cancer cells more sensitive to the anti-microtubule agent paclitaxel [7], a phenomenon consistent with the findings of Schmidt et al. [8]. Taken together, we speculate that patients with high levels of TACC3 may benefit more from radiotherapy, PARP inhibitor therapy, or a combination of both than those with comparatively lower levels of TACC3. On the other hand, patients with low levels of TACC3 may have a better response to paclitaxel. In addition to opening up new avenues for patient stratification, TACC3 also harbors prognostic potential, especially in light of aforementioned evidence of TACC3's role in EMT. Thus, tracking the status of TACC3 levels may also offer an opportunity for monitoring tumor progression and metastasis. As the emerging importance of TACC3 for cancer is increasingly apparent, we are better positioned to strategically address knowledge gaps in cancer etiology, and more effectively reap the harvest of personalized cancer therapy.
